# Ectopically Expressed Meiosis-Specific Cancer Testis Antigen HORMAD1 Promotes Genomic Instability in Squamous Cell Carcinomas

**DOI:** 10.3390/cells12121627

**Published:** 2023-06-14

**Authors:** Jennifer Gantchev, Julia Messina-Pacheco, Amelia Martínez Villarreal, Brandon Ramchatesingh, Philippe Lefrançois, Pingxing Xie, Laetitia Amar, Hong Hao Xu, Keerthenan Raveendra, Daniel Sikorski, Daniel Josue Guerra Ordaz, Raman Preet Kaur Gill, Marine Lambert, Ivan V. Litvinov

**Affiliations:** 1 Research Institute of McGill University Health Centre, Montreal, QC H4A 3J1, Canada; 2 Department of Pathology, McGill University, Montreal, QC H4A 3J1, Canada; 3 Lady Davis Institute for Medical Research, Jewish General Hospital, Montreal, QC H3T 1E2, Canada; 4 Division of Dermatology, Department of Medicine, McGill University Health Centre, Montreal, QC H4A 3J1, Canada; 5 Faculty of Medicine, McGill University, Montreal, QC H3G 2M1, Canada; 6 Faculty of Medicine, Laval University, Quebec City, QC G1V 0A6, Canada; 7 Faculty of Science, McGill University, Montreal, QC H3A 0G5, Canada

**Keywords:** HORMAD1, STRA8, meiomitosis, squamous cell carcinoma, genomic instability, meiosis-specific, cancer testis gene, meiCT, etoposide, drug resistance, carcinogenesis, DNA damage

## Abstract

Genomic instability is a prominent hallmark of cancer, however the mechanisms that drive and sustain this process remain elusive. Research demonstrates that numerous cancers with increased levels of genomic instability ectopically express meiosis-specific genes and undergo meiomitosis, the clash of mitotic and meiotic processes. These meiotic genes may represent novel therapeutic targets for the treatment of cancer. We studied the relationship between the expression of the meiosis protein HORMAD1 and genomic instability in squamous cell carcinomas (SCCs). First, we assessed markers of DNA damage and genomic instability following knockdown and overexpression of HORMAD1 in different cell lines representing SCCs and epithelial cancers. shRNA-mediated depletion of HORMAD1 expression resulted in increased genomic instability, DNA damage, increased sensitivity to etoposide, and decreased expression of DNA damage response/repair genes. Conversely, overexpression of HORMAD1 exhibited protective effects leading to decreased DNA damage, enhanced survival and decreased sensitivity to etoposide. Furthermore, we identified a meiotic molecular pathway that regulates HORMAD1 expression by targeting the upstream meiosis transcription factor STRA8. Our results highlight a specific relationship between HORMAD1 and genomic instability in SCCs, suggesting that selectively inhibiting HORMAD1, possibly, through STRA8 signaling, may provide a new paradigm of treatment options for HORMAD1-expressing SCCs.

## 1. Introduction

Cutaneous squamous cell carcinomas (cSCCs) arise from the malignant transformation of keratinocytes derived from the interfollicular epidermal layer and hair follicle stem cells [1]. cSCCs, such as other organ SCCs, bear an accumulation of mutations in genes related to cell cycle and DNA repair mechanisms that lead to genomic instability [2]. These cancers bear high mutational burdens with ~50 mutations per megabase pair of DNA [3]. Approximately 90% of cSCCs possess UV signature mutations (C→T at a dipyrimidine site, with ≥5% CC→TT), a leading cause of mutations in this cancer type.

Erroneous DNA damage responses (DDR) in cancer results in the selection of nonconventional mechanisms of repair in surviving cells that likely involve alternative classes of proteins. Emerging research suggests that the ectopic expression of a group of cancer/testis antigens (CTAs) is important in mitigating genomic instability in cancers [4,5]. CTAs are genes expressed exclusively in the testis, ovaries, and placenta, and heterogeneously expressed in various cancers [6,7]. One of >80 families of CTAs is the meiosis-specific subgroup, termed meiosis-specific cancer/testis antigens (meiCT) genes/proteins [5,8,9]. MeiCT proteins regulate meiosis initiation (STRA8), DNA Double-Strand Breaks (DSBs) (SPO11, PRDM9), components of the synaptonemal complex (SYCP1, SYCP3), homologous recombination (HORMAD1, HORMAD2, MND1, DMC1, HOP2), and cohesion (STAG3). MeiCT genes/proteins are ectopically expressed in a wide variety of cancers including breast, colorectal, stomach, brain, hematological cancers, hepatocellular, pancreatic, gastric, and skin [10]. In many cases their expression is associated with a worse clinical course and cancer progression [5,9,10].

The prevalent expression of meiCT genes across cancers and tumor types suggests that tumor cells undergo a process called meiomitosis [5,10]. Meiomitosis is defined as the coexisting activation of meiotic and mitotic proteins in a cancer cell that provides a selective advantage [11]. However, the roles of these ectopically expressed meiCT proteins in carcinogenesis remain largely elusive. Research suggests that aberrant expression and activity of meiosis proteins in cancer is crucial for chromosomal instability [5,9,12,13] due to their putative functions in DSB formation, chromosome exchange, and chromatid cohesion in meiosis [12]. Interestingly, cancers with robust expression of meiCT genes/proteins exhibit increased genomic instability [12,14,15]. This is especially the case with HORMA domain-containing protein 1 (HORMAD1; CT46) expressing cancers. In meiosis, HORMAD1 is associated with the formation and stability of the synaptonemal complex [16,17], the formation of DSBs by enabling the accumulation of DSB machinery [18,19], and enabling the accumulation and activation of the DNA damage response kinase, ATR, on unsynapsed axes [20,21]. Its HORMAD1’s HORMA-domain that interacts with short sequence motifs called ‘closure motifs’ [22,23], at their C-terminus [24], enabling HORMAD1 to self-assemble in a head-to-tail manner that contributes to its DSB and crossover functions [25]. Additionally, the HORMA domain is a conserved adaptor protein involved in the protein recruitment and repair of DNA adducts, DSBs, and non-attachment to spindles from yeast to humans [22].

HORMAD1 is regarded as a potentially important oncogene that contributes to genomic instability [12,14] and poor patient prognosis [26,27]. In lung adenocarcinoma tumors, high expression of HORMAD1 correlates with elevated mutational burden and reduced survival compared to tumors with low expression of HORMAD1 [27]. Furthermore, HORMAD1 has been shown to be involved in DNA damage repair processes such as non-homologous end joining [15], homologous recombination [27,28], and mismatch repair in breast and lung carcinomas [29]. In this study, we investigated the relationship between HORMAD1 expression in genomic instability in SCCs.

## 2. Materials and Methods

### 2.1. Cell Lines and Tissue Collection

A431 (CRL-1555; RRID:CVCL_0037), CAL27 (CRL-2095; RRID:CVCL_1107), CaSki (CRL-1550; RRID:CVCL_1100), H23 (CRL-5800; RRID:CVCL_1547), Calu6 (HTB-56; RRID:CVCL_0236), and UPCI:SCC154 (CRL-3241; RRID:CVCL_2230) cell lines were purchased from ATCC (Manassas, VA, USA). A431, CAL27, and Calu6 were cultured in Dulbecco’s Modified Eagle’s Medium (DMEM, #30-2002). CaSki and H23 were cultured in RPMI 1640 (#30-2001). UPCI:SCC154 was cultured in Eagle’s Minimal Essential Medium (EMEM, #30-2003) supplemented with 2 mM L-glutamine. Cell culture medium was obtained from ATCC (Manassas, VA, USA). Cells were supplemented with 10% fetal bovine serum (FBS, #12484028) and 5% penicillin-streptomycin (#15140122) and maintained at 37 °C with 5% CO_2_ [30,31]. Cells were periodically evaluated for mycoplasma contamination by DAPI stain for extra-nuclear DNA and MycoFluor^TM^ Mycoplasma detection kit (#M7006). Reagents listed were obtained from Thermo Fisher Scientific (Waltham, MA, USA). Human subjects in this study were patients with histopathologically-verified SCCs. This study was approved by Institutional Review Board, The Ottawa Hospital (IRB Protocol # 20150896-01H) and Research Institute of the McGill University Health Centre (IRB Protocols # 2018-4128 and 2022-8414).

### 2.2. Reagents

Etoposide (341205) was purchased from Millipore Sigma, Burlington, MA, USA.

### 2.3. shRNA-Mediated Knockdown

GIPZ HORMAD1-GFP (RHS4531-EG8407) and GIPZ STRA8-GFP (RHS4531-EG346673) shRNA constructs (Table S1) were obtained from Dharmacon Thermo Scientific (Chicago, IL, USA). Plasmids were harvested from bacterial culture using a CompactPrep Plasmid Maxi Prep (#12863) from Qiagen (Germantown, MD, USA), as per the manufacturer’s instructions. Plasmids were transfected into cell lines using DharmaFECT kb DNA transfection reagent (#T-2006-01, Dharmacon Thermo Scientific) according to the manufacturer’s instructions. Cultured cells were stably selected with 0.25–0.50 μg/mL puromycin for 2 weeks before colonies were lifted and expanded in culture. Four HORMAD1 and 6 STRA8 shRNA constructs were transfected into cells. Construct 3 and 4 for HORMAD1 and constructs 1 and 3 for STRA8 were selected to perform experiments (Figure S1).

### 2.4. LentiORF Overexpression

The open reading frame (ORF) lentiviral vector for HORMAD1 was obtained from Origene (RC207969L3V). Overexpression was achieved by transducing cancer cells with HORMAD1 lentiviral particles and polybrene (#TR-1003-G, Millipore Sigma). Cells were selected with puromycin for 2 weeks and a pooled population was used for experimentation. Western blot analysis was routinely performed to confirm HORMAD1 overexpression [32,33].

### 2.5. Immunofluorescence

Immunofluorescence staining and chromatin bridge analysis, were performed, as previously described [34]. Primary antibodies included: *γ*-H2AX (1:1000) (Abcam (Cambridge, UK), ab124781; RRID:AB_10971675), Ki67 (1:400) (Invitrogen (Carlsbad, CA, USA), PA5-16785; AB_11000602) or Pericentrin (1:200) (Invitrogen, PA5-53498; RRID:AB_2645391). Secondary antibody included: Alexa594-conjugated goat anti-rabbit (1:1000) (Cell Signaling (Danvers, MA, USA), 8889; RRID:AB_2716249). To evaluate immunofluorescence staining for markers of proliferation and instability, 500 cells for each condition and time point were used to determine the percentage of positively stained cells. Experiments/cell counts were performed on three biological replicates.

### 2.6. Cytokinesis Block Micronucleus Assay (CBMN)

CBMN was performed as previously described [34]. Three biological replicates were used to analyze the data.

### 2.7. Chromatin Bridge Analysis

The analysis of chromatin/anaphase bridges was performed as previously described [34]. Briefly, 100 anaphase/cytokinesis cells were analyzed for each condition in triplicate and were scored on whether or not a bridge was present.

### 2.8. Western Blot Analysis

Cells were lysed using RIPA buffer plus a protease and phosphatase inhibitor cocktail (#A32959, Thermo Fisher Scientific). Protein concentration was quantified using Bradford assay (#23236) from BioRad, Hercules, CA, USA. A total of 15 μg of protein was subjected to immunoblot analysis. Protein was separated by SDS/PAGE and transferred to PVDF membrane using the Trans Blot Turbo System (BioRad). Membranes were blotted with the following antibodies: HORMAD1 (1:1000) (Abcam, 178432; RRID:AB_11042521), STRA8 (1:1000) (Abcam, ab217380; RRID:AB_945678), GAPDH (1:5000) (Invitrogen, MA5-15738; RRID: AB_10977387). Target proteins were detected using Clarity Western ECL Substrate (#1705061) before being visualized with a Chemidoc Touch Imaging System (#12003153) from BioRad [35].

### 2.9. Cell Proliferation Assay

The effects of genetic manipulation of HORMAD1 and drug treatment on cell proliferation were measured using the Beckman Coulter Vi-Cell XR cell viability analyzer that uses the trypan blue dye exclusion method to evaluate cell viability (Beckman Coulter, IN, USA). Briefly, triplicates of 3 × 10^5^ cells/well were plated in a 6-well plate and cell proliferation was measured every 24 h, up to 72 h [36]. Cell proliferation was analyzed by collecting cells from the well and placing them into the Vi-Cell counter to be counted. Raw cell number data were used to graph cell proliferation over time from three technical replicates and from three biological replicates.

Multiskan Go spectrometer (T

### 2.10. Immunohistochemistry

Immunohistochemistry was performed as previously described [9,37]. Samples were incubated with HORMAD1 rabbit polyclonal antibody (Novus Biologicals, Centennial, CO, USA, NBP1-85401) or STRA8 (Abcam, ab49602; RRID:AB_945678) at a dilution of 1:500. Immunoreactivity was evaluated in Quantitative Pathology and Bioimage Analysis (Qupath; RRID:SCR_018257) using images of samples scanned on a Zeiss Axio scanner. Cell counts were performed randomly in three regions. The percentage of HORMAD1 or STRA8 immunostaining was used for analysis across 18 samples with corresponding positive and negative controls.

### 2.11. Colony Formation Assay

Cells were seeded into a 6-well plate (1 × 10^3^ cells/well) and were allowed to adhere overnight. Media was replaced with growth media supplemented with etoposide for 24 h, then replaced with fresh complete media (without a drug). Cells were cultured for 7–10 days. The resulting colonies were fixed with glacial acetic acid, stained with 0.1% crystal violet, and colonies were counted using ImageJ 1.6 software (National Institutes of Health, Bethesda, MD, USA). Clonogenic assays were performed on 3 biological replicates.

### 2.12. TCGA Data

Data derived from 23 cancers were downloaded from The Cancer Genome Atlas, RRID:SCR_003193 (TCGA) database http://cancergenome.nih.gov (accessed on 10 June 2021). Statistical analysis was performed by using Bayesian statistics with a non-informative prior to compare differential gene expression between normalized transcripts per million (TPM) values of tumor and normal adjacent tissues [9].

### 2.13. RNA-Seq Data Analysis

Raw data were obtained from the McGill Genome Centre in FASTQ format. RNA read quality was assessed using FastQC 0.11.9 [38] and the Trimmomatic 0.39 tool [39] was used to preprocess the data [39]. The reads were aligned, and transcripts were quantified using Kallisto 0.48.0 software [40]. The human genome GRCh38 and annotation from ENSEMBL (Homo sapiens version 105) were used to create the transcriptome annotation. Gene counts and gene TPM were obtained by summing the corresponding value of each transcript of a gene. The differential expression was performed using the DESeq2 1.34.0 R package [41], and the pseudocounts were imported using the tximport 1.22.0 package [42]. Pathway enrichment analysis was performed using gprofiler [43].

### 2.14. Statistical Analysis

Quantitative results were obtained from a minimum of three independent experiments. Data were analyzed using the GraphPad Prism 9 software package (GraphPad Prism, San Diego, CA, USA, RRID:SCR_002798). Differences between means of three biological replicates were determined by either the Student’s *t*-test or by two-way ANOVA and Tukey’s multiple comparisons. Means were considered statistically significant at *p* < 0.05 [44,45].

## 3. Results

### 3.1. HORMAD1 Expression Is Significantly Increased in SCCs

**Figure 1:**
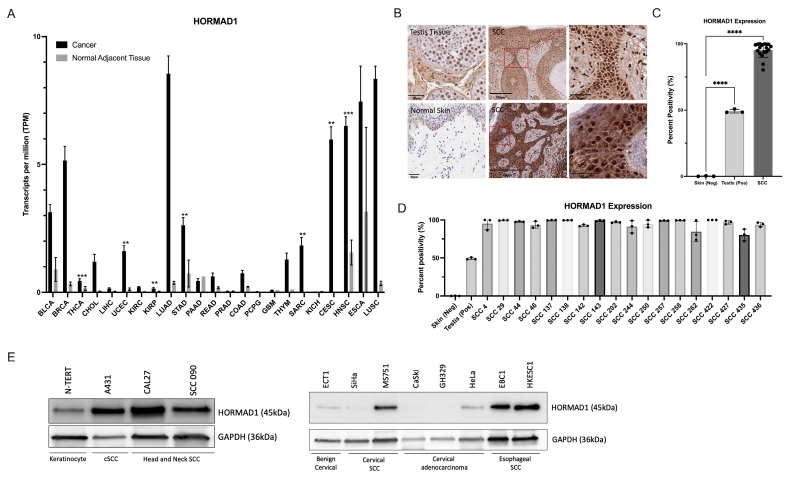
Significant increase in the ectopic expression of HORMAD1 in Squamous Cell Carcinomas (SCCs). (**A**) Bar graph of TCGA data detailing average transcripts per million (TPM) for HORMAD1 gene expression across 23 cancers. A significant increase in HORMAD1 expression is observed in squamous cell carcinomas (SCCs) of the cervix (CESC), head and neck (HNSC), esophagus (ESCA), and lung (LUSC) compared to normal adjacent tissue. (**B**) Immunohistochemical analysis of HORMAD1 protein expression in patient biopsy samples of cSCC. HORMAD1 positive control staining (normal human testis) is presented in the upper left panel and normal skin staining is presented in the lower left panel. The remaining panels are cSCC tissues with corresponding magnification presented in the upper and lower right panels. (**C**) Quantification of HORMAD1 expression in 18 cSCC patient biopsy samples compared to normal skin and human testis. (**D**) Bar graph detailing HORMAD1 protein expression in each patient biopsy sample. (**E**) Qualitative expression of HORMAD1 in cell lines representing benign keratinocyte cell line N-TERT, cutaneous SCC cell line, A431, head and neck SCC cell lines CAL27 and SCC090, benign cervical cell line ECT1, cervical and esophageal SCCs, and cervical adenocarcinoma. Sample loading was completed per cell number, not based on protein concentration. Values are means ± SEM, n = 3, **** *p* > 0.0001, *** *p* > 0.001, ** *p* > 0.01; BLCA (Bladder Urothelial Carcinoma); BRCA (Breast invasive carcinoma); THCA (Thyroid carcinoma); CHOL (Cholangiocarcinoma); LIHC (Liver hepatocellular carcinoma); UCEC (Uterine Corpus Endometrial Carcinoma); KIRC (Kidney renal clear cell carcinoma); KIRP (Kidney renal papillary cell carcinoma); LUAD (Lung adenocarcinoma); STAD (Stomach adenocarcinoma); PAAD (Pancreatic adenocarcinoma); READ (Rectum adenocarcinoma); PRAD (Prostate adenocarcinoma); COAD (Colon adenocarcinoma); PCPG (Pheochromocytoma and Paraganglioma); GBM (Glioblastoma multiforme); THYM (Thymoma); SARC (Sarcoma); KICH (Kidney Chromophobe).

We analyzed *HORMAD1* mRNA expression across 23 cancer subtypes using TCGA data paired with normal adjacent tissue samples. Our results revealed notably elevated *HORMAD1* transcription expression in all but four cancer tissues compared to their corresponding normal adjacent tissue samples (Figure 1A). Notably, there was a marked enhanced expression across SCCs, including cervical SCC and endocervical adenocarcinoma (CESC), head and neck squamous cell carcinoma (HNSC), esophageal squamous cell carcinoma (ESCA), and lung squamous cell carcinoma (LUSC) compared to other cancer types.

Unfortunately, cSCC data was not available in the TCGA dataset. Therefore, to determine HORMAD1 protein localization and expression patterns in cSCC, we performed immunohistochemical analysis of 18 cSCC tumor biopsy samples isolated from patients. All 18 samples demonstrated strong nuclear staining and diffuse cytoplasmic staining of HORMAD1 in pleomorphic squamous cells invading the dermis (Figure 1B,D). Our results demonstrate that the percentage expression of HORMAD1 in all 18 cSCCs analyzed was significantly higher (95%) compared to normal skin (0%) and human testis (49%) (Figure 1C,D). Lastly, we examined relative HORMAD1 levels in various SCC cell lines (cutaneous, head and neck, cervical and esophageal) to identify HORMAD1-positive and HORMAD1-negative cells. This information provided insight into cell lines that were appropriate for use in further experimentation (Figure 1E).

### 3.2. HORMAD1 Influences DNA Damage and Genomic Instability in SCC Cells

**Figure 2:**
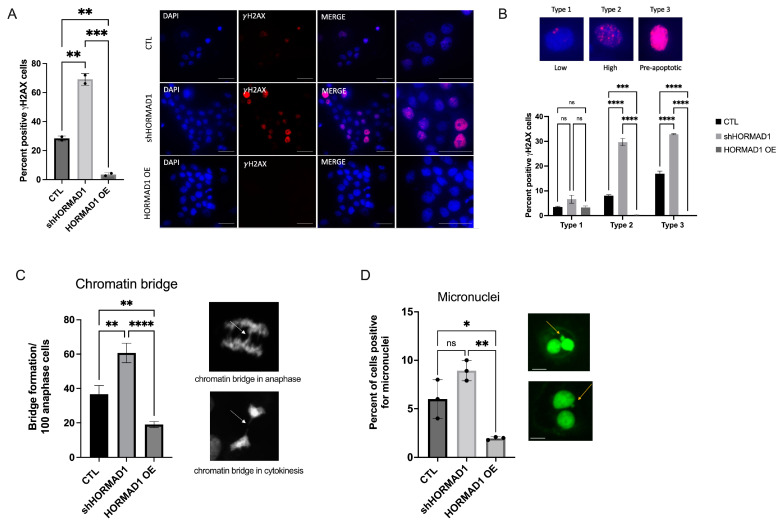
HORMAD1 expression influences DNA damage and genomic instability in the cSCC cell line, A431. (**A**) shRNA-mediated knockdown of HORMAD1 (shHORMAD1) results in increased *γ*H2AX staining (red) indicating high levels DSBs in cells counterstained with DAPI (blue), while overexpression of HORMAD1 (HORMAD1 OE) exhibits minimal *γ*H2AX staining compared to non-silencing CTL cells. Corresponding representative immunofluorescent *γ*H2AX staining for CTL, shHORMAD1 and HORMAD OE cells. Scale bars represent 50 μm. (**B**) *γ*H2AX (magenta) staining separated into 3 staining types corresponding to the degree of DNA damage: type 1, low DNA damage; type 2, high DNA damage; and type 3, preapoptotic cells (upper panel). Magnification 1000×. When percent positive *γ*H2AX cells are separated into respective types, shHORMAD1-treated cells display high degree of type 2–3 *γ*H2AX staining (high DNA damage and preapoptotic cells), whereas HORMAD1 OE cells have low levels of DNA damage demonstrated primarily by type 1 *γ*H2AX staining. (**C**) shHORMAD1 cells exhibit increased genomic instability as indicated by an elevated number of chromatin bridges (arrows, magnification 1000×) in anaphase and cytokinesis, and a significant increase in (**D**) micronuclei formation (arrows) in cells, nucleic acid stained with cytochalasin B (2 μg/mL) (green). A decrease in chromatin bridge and micronuclei formation was found in HORMAD1 OE cells. Scale bars represent 100 μm. Values are means ± SEM, n = 3, **** *p* > 0.0001, *** *p* > 0.001, ** *p* > 0.01, * *p* > 0.1, ns (not significant); SCC (squamous cell carcinoma); OE (overexpression).

Given that HORMAD1 modulates homologous recombination during meiosis in mice [46,47], we tested whether the amount of endogenous DSBs would change if HORMAD1 protein expression was altered in a HORMAD1 expressing SCC cell lines. We examined the impact of HORMAD1 knockdown and overexpression (Figure S1) on DNA damage in the cSCC cell line, A431. First, our results demonstrated that *γ*H2AX staining robustly increased following shRNA-mediated knockdown of HORMAD1 compared to control non-silencing cells (CTL), indicating an increase in DSBs (Figure 2A). Conversely, *γ*H2AX staining was significantly decreased in HORMAD1 overexpressing (HORMAD1 OE) A431 cells, suggesting a protective effect of HORMAD1 expression against DNA damage (Figure 2A). To differentiate the severity of damage between shHORMAD1, HORMAD1 OE, and CTL, *γ*H2AX staining was classified into three types: type 1 with <10 foci indicative of low DNA damage; type 2 with >10 foci indicative of high DNA damage; and type 3 with pan nuclear staining indicative of pre-apoptotic cells, as detailed in [48]. We observed that shHORMAD1-treated cells had significantly more type 2 and type 3 *γ*H2AX staining indicating high levels of DNA damage and pre-apoptotic cells whereas, HORMAD1 OE cells demonstrated low level of DNA damage, primarily, type 1 *γ*H2AX staining (Figure 2B). To corroborate these findings, we also investigated 53BP1 staining [34] and found similar increases in moderate levels of DNA damage indicated by type 2 53BP1 foci staining in shHORMAD1-treated cells compared to CTL (Figure S2).

To investigate other components of DNA damage, we stained synchronized A431 cells with DAPI immunofluorescence to evaluate chromatin bridge and micronuclei formation, common indicators of genomic instability [34]. Chromatin bridge formation occurs when fused chromosomes are pulled towards opposing poles during mitosis [49], the presence of persistent intermediates of recombination repair, during incomplete replication of chromosomal loci, and when chromosomes become intertwined [50]. Consistent with the *γ*H2AX staining, chromatin bridge formation was significantly higher in shHORMAD1 cells and decreased in HORMAD1 OE cells compared to CTL cells (Figure 2C), indicating that HORMAD1 influences the level of genomic instability and DNA damage in SCC cells.

Micronuclei are isolated nuclear structures encased in their own nuclear envelope outside of the main nucleus. They are a robust marker of genomic instability created by lagging chromosomes [34], DNA damage, and mitotic errors [51]. We used the cytokinesis block micronucleus assay (CBMA) [52] to assess micronuclei formation related to HORMAD1 expression. Micronuclei formation increased significantly in shHORMAD1 cells and decreased in HORMAD1 OE cells when compared to CTL cells (Figure 2D). Taken together, these results suggest that DNA damage and genomic instability are significantly enhanced when HORMAD1 is depleted in HORMAD1 expressing SCC cells, while HORMAD1 overexpression provides protection from DNA damage (Figure 2A–D). Comparable results were obtained in the SCC cell line, CAL27, and in epithelial SCC cell lines (CaSki, Calu6, H23) (Figure S3).

### 3.3. HORMAD1 Knockdown Leads to Reduced Proliferation and Survival in SCC Cells

**Figure 3:**
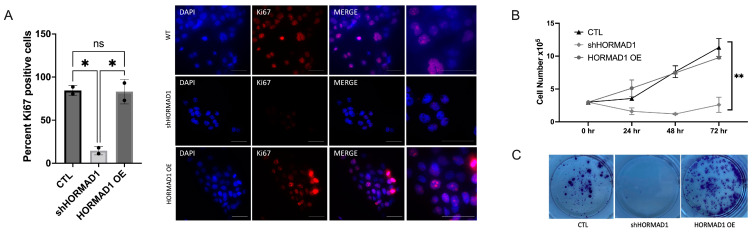
Depletion of HORMAD1 leads to decreased proliferation and survival. (**A**) Percent of Ki67 positive cells (red) in control non-silencing cells (CTL), shHORMAD1 and HORMAD1 OE A431 cells 24 h following plating. Nuclei counterstained with DAPI (blue). Scale bars represent 50 μm. (**B**) Consistent with Ki67 staining, proliferation of shHORMAD1 cells significantly decreased 24, 48, and 72 h after plating. (**C**) Survival/clonogenic assay results complement proliferation results, shHORMAD1 cells formed few colonies, while HORMAD1 OE cells formed significantly more colonies than CTL cells. Values are means ± SEM, n = 3, ** *p* > 0.01, * *p* > 0.1, ns (not significant), SCC (squamous cell carcinoma); OE (overexpression).

HORMAD1 expression influences DNA damage and could therefore influence proliferative potential and survival. To test this hypothesis, we performed a cell count assay immunofluorescence staining for Ki67 and a clonogenic assay. Overexpression of HORMAD1 in A431 cells had no effect on Ki67 staining (Figure 3A), nor cell counts (Figure 3B) compared to control, signifying that higher expression of HORMAD1 does not enhance proliferation in cells that already express HORMAD1 protein. Interestingly, overexpression of HORMAD1 did enhance survival, as indicated by clonogenic assays (Figure 3C). Conversely, when HORMAD1 was depleted, Ki67 staining and cell proliferation decreased significantly, and survival was impaired (Figure 3A–C). It is likely that the significant presence of DNA damage in knockdown cells leads to impairments in proliferation and survival. Similar results were obtained in other SCC and adenocarcinoma cell lines (Figure S3).

**Figure 4:**
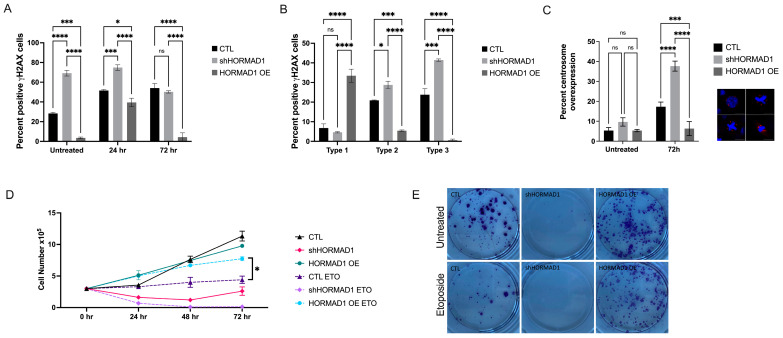
HORMAD1 expression provides protection/resistance against DNA damage following etoposide treatment. (**A**) Quantitative immunofluorescence cell count analysis documenting percent *γ*H2AX staining in non-silencing CTL cells, shHORMAD1 and HORMAD1 OE A431 cells treated with 1 μM etoposide (24 and 72 h panels). At 24 and 72 h following etoposide treatment, shHORMAD1 cells had a significantly higher percentage of *γ*H2AX positive cells, while HORMAD1 OE cells had a significantly lower percentage of *γ*H2AX positive cells in comparison to CTL. (**B**) Distribution of percent *γ*H2AX positive cells by corresponding DNA damage type (type 1—low, type 2—high, type 3—preapoptotic, based on *γ*H2AX staining pattern) in cells treated with 1 μM etoposide for 24 h. (**C**) Percentage of centrosome amplification, a marker of genomic instability, indicated by pericentrin immunofluorescence staining (red) in cells treated with etoposide for 72 h. Nuclei counterstained with DAPI (blue). shHORMAD1 cells exhibit centrosome amplification, whereas HORMAD1 OE cells demonstrate a significantly lower percentage amplification compared to CTL. Example images are presented. Magnification 1000×. (**D**) Proliferation assays evaluating cell number over 72 h in untreated and etoposide-treated A431 cells. Untreated shHORMAD1 cells exhibit decreased proliferation that is further inhibited following etoposide treatment. Proliferation of HORMAD1 OE cells is minimally affected by etoposide treatment. (**E**) Clonogenic assays measuring cell survival in untreated and in 1 μM etoposide treated cells over 7–10 days. Values are means ± SEM, n = 3, **** *p* > 0.0001, *** *p* > 0.001, * *p* > 0.1, ns (not significant); SCC (squamous cell carcinoma); OE (overexpression).

The accurate repair of DNA damage, particularly of DSBs, is vital for sustaining genome integrity. Defective DNA repair and enhanced instability are considered important contributors of carcinogenesis and lead to the chromosomal abnormalities (i.e., inversions, deletion, and translocations) seen in aggressive tumors [53]. We examined treatment sensitivity in HORMAD1 overexpressing and HORMAD1-depleted SCC cells following treatment with etoposide, a topoisomerase II inhibitor that induces DNA damage. HORMAD1 knockdown resulted in a significant increase in *γ*H2AX staining following etoposide treatment (Figure 4A). At 24 h, shHORMAD1 cells exhibited predominantly type 3 *γ*H2AX staining, indicative of pre-apoptotic cells (Figure 4B). Although HORMAD1 overexpressing cells demonstrated a slight increase in overall damage 24 h following etoposide treatment (Figure 4A), the *γ*H2AX staining was exclusively type 1, indicating a low level of damage when compared to a type 2 staining pattern (Figure 4B and Figure S4). These results signify that HORMAD1 depletion in HORMAD1 expressing SCC cells results in an increased sensitivity to etoposide, while overexpression of HORMAD1 prevents the acquisition of high DNA damage. Similar results were obtained in other SCC cell lines (Figure S5).

Subsequently, we verified if there was a relationship between HORMAD1 expression levels and other indicators of genomic instability following etoposide treatment. Consistent with our results demonstrating increased genomic instability in shHORMAD1 cells, etoposide treatment led to an increased expression of centrosomes, indicated by aberrant pericentrin staining (Figure 4C).

To evaluate the proliferation of shHORMAD1 and HORMAD1 OE cells following etoposide treatment, we performed a cell count assay following 72 h of treatment. Control and shHORMAD1 cells exhibited decreased proliferation in as little as 24 h following etoposide treatment. Interestingly, HORMAD1 OE cells decreased proliferation compared to control following of etoposide treatment but demonstrated an increasing proliferation trend after 72 h of treatment (Figure 4D). These results highlight a protective role of HORMAD1 expression likely due to its ability to participate in a DNA damage response. Consistently, clonogenic assays demonstrated decreased survival in shHORMAD1 cells and enhanced survival in HORMAD1 OE cells compared to control (Figure 4E). Similar results were obtained using other SCC cell lines (Figure S6).

### 3.4. HORMAD1 Expression Is Regulated by the Meiosis-Specific Transcription Factor STRA8 in SCC Cells

**Figure 5:**
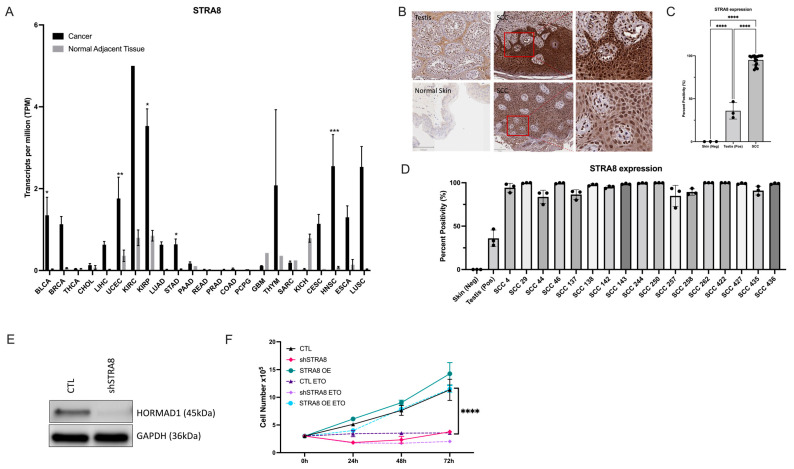
STRA8 is ectopically expressed in SCCs and its inhibition downregulates HORMAD1. (**A**) Bar graph of TCGA analysis documenting average transcripts per million (TPM) for *STRA8* gene expression across 23 cancers. Consistent with *HORMAD1* expression, *STRA8* is significantly upregulated in squamous cell carcinomas (SCCs) of the cervix (CESC), head and neck (HNSC), esophagus (ESCA), and lung (LUSC) compared to normal adjacent tissue. (**B**) Immunohistochemical analysis of STRA8 protein expression in patient biopsy samples of cSCCs. STRA8 positive control (normal human testis) is presented in the upper left panel and normal skin biopsy staining is presented in the lower left panel (scale bars represent 50 μm). The remaining panels show STRA8 staining in cSCC tissues with respective magnification (red square in middle panels; scale bars represent 250 μm) presented in upper and lower right panels (scale bars 50 μm). (**C**) Quantification of STRA8 expression in 18 cSCC patient biopsy samples compared to normal skin. (**D**) Bar graph detailing STRA8 protein expression in each patient biopsy sample in our patient cohort (**C**). (**E**) Immunoblot representing the diminished expression of HORMAD1 protein following shRNA-mediated knockdown of STRA8 (construct 1—Figure S1D) in A431 cells. (**F**) Cell proliferation results over 72 h for CTL, shSTRA8, and STRA8 OE A431 cells in the presence or absence of 1 μM etoposide treatment. Values are means ± SEM, n = 3, **** *p* > 0.0001, *** *p* > 0.001, ** *p* > 0.01, * *p* > 0.1. OE (overexpression); BLCA (Bladder Urothelial Carcinoma); BRCA (Breast invasive carcinoma); THCA (Thyroid carcinoma); CHOL (Cholangiocarcinoma); LIHC (Liver hepatocellular carcinoma); UCEC (Uterine Corpus Endometrial Carcinoma); KIRC (Kidney renal clear cell carcinoma); KIRP (Kidney renal papillary cell carcinoma); LUAD (Lung adenocarcinoma); STAD (Stomach adenocarcinoma); PAAD (Pancreatic adenocarcinoma); READ (Rectum adenocarcinoma); PRAD (Prostate adenocarcinoma); COAD (Colon adenocarcinoma); PCPG (Pheochromocytoma and Paraganglioma); GBM (Glioblastoma multiforme); THYM (Thymoma); SARC (Sarcoma); KICH (Kidney Chromophobe).

We sought to investigate upstream regulators of HORMAD1 to determine if there are indirect targets that impact HORMAD1 expression in SCC cells. In mouse testis, *Hormad1* transcription is regulated by the transcription factor stimulated by retinoic acid 8 (STRA8) at the onset of meiosis [54]. TCGA analysis revealed a marked increase of STRA8 in SCCs (CESC, HNSC, ESCA, LUSC) (Figure 5A), consistent with results found with *HORMAD1* (Figure 1A). Furthermore, 18 cSCC patient biopsy samples stained for HORMAD1 in Figure 1B were concomitantly stained for STRA8. STRA8-positive staining was observed in 95.15% of nuclei of pleomorphic squamous cells invading the dermis compared to normal skin (0%) and human testis (35.93%) (Figure 5B–D). Interestingly, shRNA-mediated knockdown of STRA8 (shSTRA8) resulted in a decrease in HORMAD1 protein expression (Figure 5E).

To determine if genetic manipulation of STRA8 in A431 cells also influences proliferation in a similar manner to HORMAD1 overexpression/knockdown, we performed a cell count proliferation assay. Depleting STRA8 (shSTRA8) resulted in decreased proliferation, while STRA8 overexpression (STRA8 OE) led to a slight increase in proliferation up to 72 h following cell plating (Figure 5F). Lastly, we performed a proliferation assay in untreated and etoposide treated CTL, shSTRA8 and STRA8 OE A431 cells. Consistent with the hypothesis that STRA8 regulates *HORMAD1* transcription/expression, STRA8 overexpression resulted in minimal changes in proliferation despite treatment with etoposide. The proliferation of STRA8 OE cells treated with etoposide was comparable to CTL untreated A431 cells. In contrast, shSTRA8 cells exposed to etoposide demonstrated significantly decreased proliferation (Figure 5F).

### 3.5. HORMAD1 Expression Leads to Changes in DNA Repair Gene Expression

**Figure 6:**
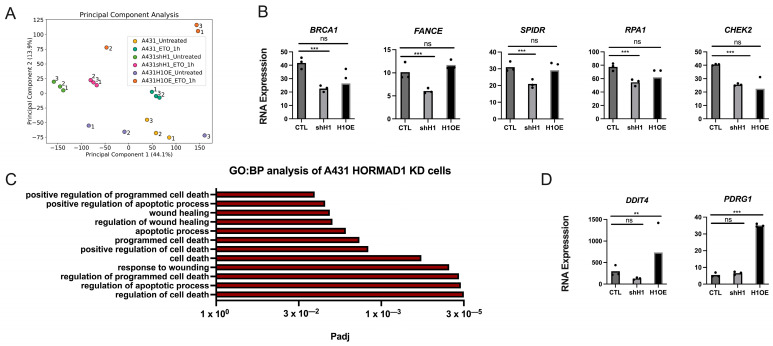
Differential expression analysis of RNA-seq data from etoposide-treated and untreated A431 cells. (**A**) Principal component analysis (PCA) plot depicts clusters of triplicate samples based on similarities in the cells. (**B**) Significantly downregulated DNA repair genes in shRNA-mediated knockdown of HORMAD1 in A431 cells (shHORMAD1 or shH1) or HORMAD1 overexpression (HORMAD1 OE or H1OE). (**C**) GO BP analysis of upregulated differentially expressed genes (DEGs) in A431 shH1 cells compared to control A431 cells. (**D**) Significantly upregulated DNA repair genes in lentiviral-mediated HORMAD1 overexpressed cells (H1OE), ns (not significant). Values are means ± SEM, n = 3, *** *p* > 0.001, ** *p* > 0.01.

To investigate transcriptional changes in DNA repair genes, we performed RNA-sequencing in untreated and etoposide-treated A431 cells. Principal component analysis (PCA) analysis demonstrated that HORMAD1 OE A431 samples do not cluster tightly and exhibit considerable variability between replicates when compared to control and shHORMAD1 cells (Figure 6A). Interestingly, HORMAD1 OE cells treated with etoposide clustered more closely to untreated control cells, corresponding to proliferation analyses that demonstrate phenotypic similarities between these conditions (Figure 3).

HORMAD1 knockdown (shHORMAD1) resulted in a significant downregulation of key DNA repair genes involved in homologous recombination repair (BRCA1, FANCE and SPIDR), single strand break stability and repair (RPA1), and regulation of DNA damage response (CHEK2) (Figure 6B). These results indicate the likelihood of an impaired capacity to engage these signaling mechanisms following HORMAD1 depletion, leading to increased etoposide sensitivity. Additionally, the downregulated expression of CHEK2 suggests changes in cell cycle regulation. However, we did not observe any significant changes in cell cycle regulatory gene expression in shHORMAD1 cells (Figure S8). Gene ontology (GO) was used to identify biological processes (BP) of differentially expressed genes (DEGs) in shHORMAD1 cells compared to control (CTL) cells. Processes involving apoptosis regulation, wound healing, and cell death were significantly upregulated following HORMAD1 depletion (Figure 6C), which was consistent with the results of increased DNA damage and with decreased proliferation and survival in shHORMAD1 cells.

Lastly, HORMAD1 overexpression resulted in increased expression in DDIT4 and PDRG1 genes associated more broadly with a DNA damage repair response [55,56] as opposed to a specific repair pathway (Figure 6D). Together, these results indicate that HORMAD1 expression in shHORMAD1 cells exhibit a relationship with DNA damage response and repair.

## 4. Discussion

This study demonstrates the importance of HORMAD1 expression in different types of SCCs, with emphasis on HORMAD1’s ability to influence levels of genomic instability, attenuate etoposide-induced DNA damage, and impact cell proliferation/clonogenicity. Downregulation of HORMAD1 in SCCs cancer cells sensitized them to etoposide treatment and may sensitize HORMAD1 dependent cells/tumors to other chemotherapy treatments, as demonstrated in studies involving breast and lung adenocarcinoma models [27,28,29]. Our work also highlights the influence of the meiosis-specific transcription factor, STRA8, in regulating HORMAD1 expression. Extensive patient TCGA analysis for various SCCs (head and neck, cervix, lung, esophagus) tissues combined with our analysis of HORMAD1 expression in freshly obtained cSCCs tumors highlight the clinical relevance of this protein and its role in modulating genomic instability. Notably, the role of HORMAD1 and thus meiomitosis is not restricted to SCCs, since other cancers with high mutational burdens, including triple negative breast cancer (TNBC) and lung adenocarcinoma, also express high levels of HORMAD1 [57].

The upstream regulation of HORMAD1 expression in cancer has not been investigated. Hence, we studied its putative regulator in meiosis, STRA8 [54]. When STRA8, a transcriptional regulator of HORMAD1 in mouse preleptotene stage germ cells [54], is depleted, HORMAD1 expression decreases, demonstrating that STRA8 regulates HORMAD1 expression in cSCC. However, whether STRA8 acts as a transcription factor in this context remains to be determined. STRA8 expression is induced by retinoic acid (RA) signaling in both male and female vertebrate mammals [58,59,60]. The ectopic expression of STRA8 and RA signaling in cSCC is intriguing, since retinoids are active in the prevention of cSCCs (reviewed in [61]).

We demonstrated that HORMAD1 expression levels correlate with the magnitude of DNA damage and genomic instability. Since similar results were observed in epithelial cancer cell lines, the relationship between HORMAD1 and genomic instability may be relevant in other cancer types. The depletion of HORMAD1 results in increased DSBs, highlighting the importance of HORMAD1 in genome integrity, even in the absence of exogenous stressors such as irradiation and chemotherapy. Additionally, when CRISPR Cas9 was used to knockout HORMAD1 in cSCC cell lines A431 and SCC154, there were no surviving cells to form stable colonies. These findings suggest that HORMAD1-ectopically expressing cells recapture this gene/protein in a novel way and become dependent on HORMAD1 expression to survive. This could explain why a 100% knockdown of HORMAD1 using shRNA was not acquired in this study. Conversely, HORMAD1 overexpression had an enhanced effect in A431 and resulted in protection from genomic instability and high levels of DNA damage, with and without treatment of etoposide. These results are in line with HORMAD1’s role in enhanced DNA repair [15,28,29,62], enabling tumor cell survival and implicating HORMAD1 oncogene as a candidate for therapeutically-resistant cancers.

Cancers expressing high levels of HORMAD1 exhibit increased resistance to select treatments [57]. Recent studies have demonstrated that HORMAD1 expression is related to a resistance to docetaxel [63], radiation [28], and oxidative and genotoxic injury [27]. However, our study has demonstrated that HORMAD1 expression knockdown in SCC cell lines results in an increase in genomic instability and in a decrease in cell survival. We propose that HORMAD1 expression facilitates DNA damage repair in strenuous environments that permits a subset of HORMAD1-dependent cells to survive and clonally expand, thus when HORMAD1 is depleted, repair and survival mechanisms fail, resulting in cell death. The mechanisms and pathways that involve HORMAD1 to support this phenotype remain largely elusive.

Therapeutic resistance attributed to HORMAD1 expression is proposed to be mediated either though homologous recombination repair (HRR) [27,28,62,63] or non-homologous end joining (NHEJ) [15,63] and is dependent on replication stress pathways such as translational synthesis [64]. It should be considered that HORMAD1 may mediate both NHEJ, HR and other repair mechanisms/responses in a context-dependent manner [63]. Our RNA-sequencing results demonstrate a significant downregulation of the HRR genes BRCA1, FANCE, and SPIDR transcripts following HORMAD1 downregulation in A431 cells supporting HORMAD1’s role in HRR. We also show that RPA1, a protein involved in the stability of single strand breaks, and CHEK2, the DNA damage response regulator involved in modulating cell cycle arrest, are also significantly downregulated in shHORMAD1 cells.

Although our cell culture results indicate that increased HORMAD1 expression in HORMAD1-dependent cell lines leads to a decrease in genomic instability, the RNA transcript repertoires did not cluster closely between triplicates, a phenomenon that may support the observation that high HORMAD1-expressing cancers exhibit increased heterogeneity and poor prognosis [57]. These results suggest that DNA damage may not be the only mechanism where HORMAD1 expression can contribute to heterogeneity and remains to be explored.

Interestingly, differentially expressed genes analysis revealed that HORMAD1 OE resulted in upregulated DDIT4 and PDRG1 gene expression. DDIT4 (DNA damage-inducible transcript 4) suppresses mTORC1, regulates cell growth and tumorigenesis, and is associated with decreased expression of pro-apoptotic proteins [55]. It is also associated with advanced stages of colorectal carcinoma [55]. PDRG1 is an oncogenic protein that mediates the ATM-p53 signaling pathway and is associated with decreased differentiation, advanced disease, and metastasis in gastric and bladder cancers [65,66]. How HORMAD1 and these genes are related remains unknown but may provide a rationale as to why high HORMAD1 expressing cancers exhibit advanced heterogeneity, increased resistance to therapy, and poor clinical prognosis.

## 5. Conclusions

The ectopic expression of HORMAD1 was documented by us and others across a variety of aggressive cancers. While in meiosis HORMAD1 regulates DNA double strand break repair during chromosomal crossover, and in cancer it influences genomic instability. In this study, we demonstrated that elevated HORMAD1 attenuates detrimental genomic instability in SCCs, thereby promoting cancer cell survival. In addition, we showed that HORMAD1 protects cells from high DNA damage following etoposide treatment and increases cell proliferation/survival. Our work highlights that HORMAD1 is an intriguing novel therapeutic target for the treatment of SCCs and other aggressive cancers. One critical advantage is that HORMAD1 is not expressed in normal somatic tissues. Therefore, treatments targeting HORMAD1 will likely have a wide therapeutic window. Furthermore, investigating the function of this protein in the context of cancers will likely yield a better understanding of meiomitosis-driven genomic instability and its implications in carcinogenesis.

## Data Availability

Are available upon request from the corresponding author.

## Supplementary Materials

The following supporting information can be downloaded at: https://www.mdpi.com/article/10.3390/cells12121627/s1, Figure S1: HORMAD1 protein expression levels following shRNA mediated knockdown and lentiviral overexpression; Figure S2: Depleted HORMAD1 protein expression leads to elevated levels of Type 2 DNA damage as indicated by the 53BP1 staining; Figure S3: HORMAD1 expression influences DNA damage and genomic instability in different SCC cells; Figure S4: HORMAD1 knockdown increases sensitivity to etoposide; Figure S5: HORMAD1 depletion leads to high levels of DNA damage; Figure S6: HORMAD1 expression decreases sensitivity to etoposide; Figure S7: RNA-sequencing volcano plots; Figure S8: Cell cycle regulation RNA-sequencing transcript expression in shHORMAD1 and HORMAD1 OE A431 cells compared to CTL; Table S1: HORMAD1 and STRA8 shRNA constructs.
