# Supplementary material for: Ectopically Expressed Meiosis-Specific Cancer Testis Antigen HORMAD1 Promotes Genomic Instability in Squamous Cell Carcinomas
Source: Cells. 2023 Jun 14;12(12):1627. doi: 10.3390/cells12121627 (PMC10296909; doi:10.3390/cells12121627)
Supplement: Supplementary file 1 [file cells-12-01627-s001.zip › cells-2344214-supplementary.pdf]

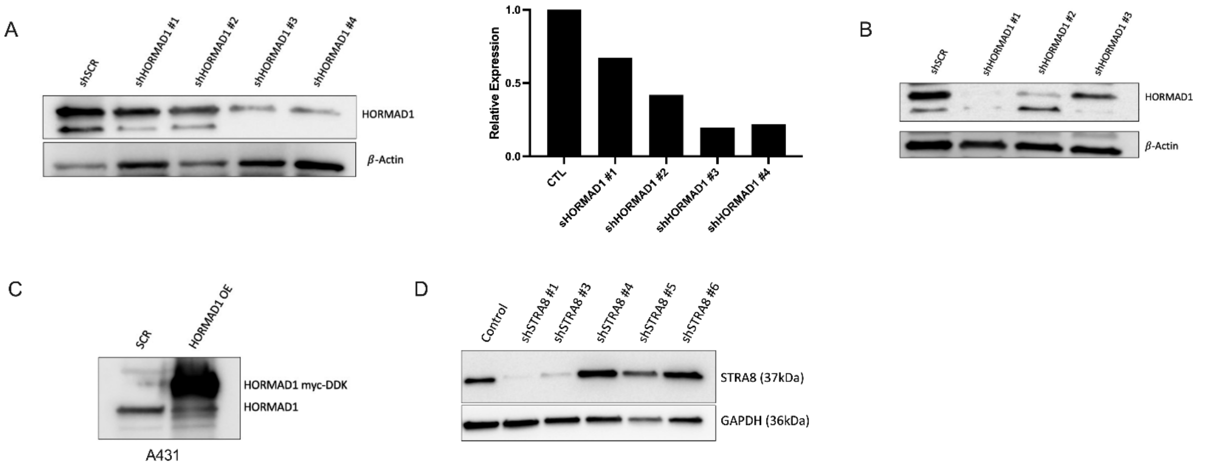

Figure S1. HORMAD1 protein expression levels following shRNA mediated knockdown and lentiviral overexpression. (A) Western blot analysis of shRNA-mediated knockdown of HORMAD1 in the cutaneous squamous cell carcinoma (cSCC) cell line, A431 with corresponding quantification of relative expression in different clones. **(B)** Western blot analysis of shRNA mediated knockdown of HORMAD1 in the head and neck SCC cell line, CAL27. **(C)** Western blot analysis of Lenti-ORF overexpression of myc-DDK tagged HORMAD1 in A431. **(D)** Western blot analysis of shRNA mediated knockdown of STRA8 in cSCC cell line, A431.

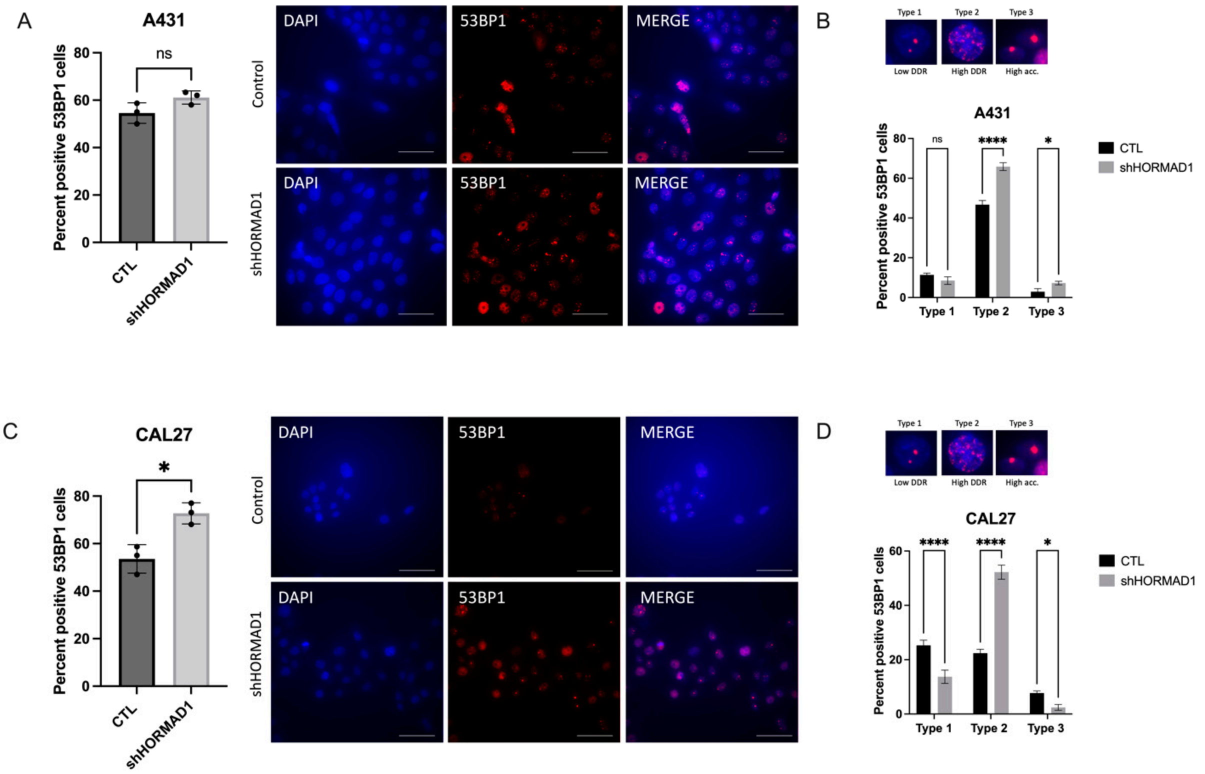

Figure S2. Depleted HORMAD1 protein expression leads to elevated levels of DNA damage as indicated by the 53BP1 staining. (A) Percentage of 53BP1 staining cells increases following shRNA mediated knockdown of HORMAD1 in A431 cSCC cells. Corresponding immunofluorescence detailing positive staining; 53BP1 (red), nucleic acid counterstain DAPI (blue). Scale bars represent 50μm. **(B)** Percentage of positive 53BP1 cells organized according to the type of DNA damage: type 1 is indicative of a low DNA damage response with one or two discreet nuclear foci; type 2 is indicative of a high DDR with three or more discrete nuclear foci; and type 3 is indicative of abnormal and heterogenous 53BP1 expression with larger (1μm) nuclear foci. Knockdown of HORMAD1 leads to elevated high DDR levels compared to control A431 cSCC cells. Magnification 1000X. **(C)** Similarly, head and neck SCC cell line, CAL27 demonstrated increased 53BP1 positive staining following shRNA mediated knockdown of HORMAD1. **(D)** Although control CAL27 cells have higher background levels of low DDR (type 1 staining), shHORMAD1 cells have significantly higher number of type 2 staining cells demonstrating high DDR. Values are means $\pm$ SEM, n=3, ****p>0.0001, *p>0.1. DDR, (DNA damage response).

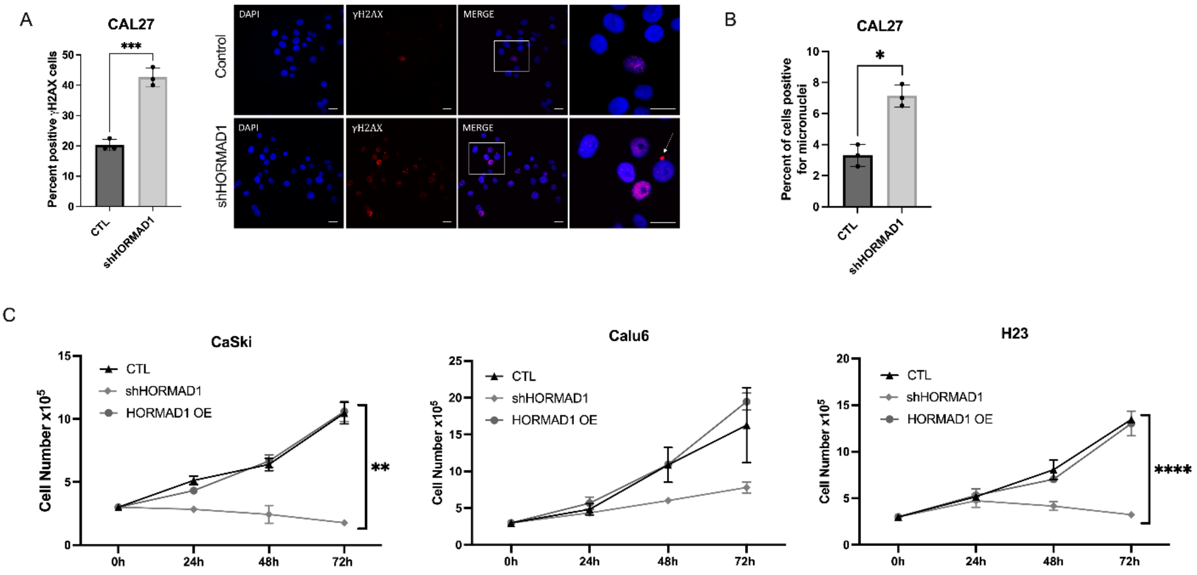

Figure S3. HORMAD1 expression influences DNA damage and genomic instability in different SCC cells. **(A)** Percent γH2AX staining (red) in CAL27 cells is significantly increased following HORMAD1 knockdown (shHORMAD1). Nucleic acid counterstained with DAPI (blue). Squares in immunofluorescence merge panels are enlarged to demonstrate γH2AX positive staining patterns. Arrows denote micronuclei. Scale bars represent 50μm. **(B)** CAL27 cells demonstrate greater percent of micronuclei formation in shHORMAD1 cells. **(C)** Proliferation assays of cell lines representing cervical adenocarcinoma (CaSki) and lung adenocarcinomas (Calu6 and H23) reveal that knockdown of HORMAD1 results in decreased proliferation. Values are means $\pm$ SEM, n=3, ****p>0.0001, ***p>0.001, **p>0.01, *p>0.1. OE (overexpression).

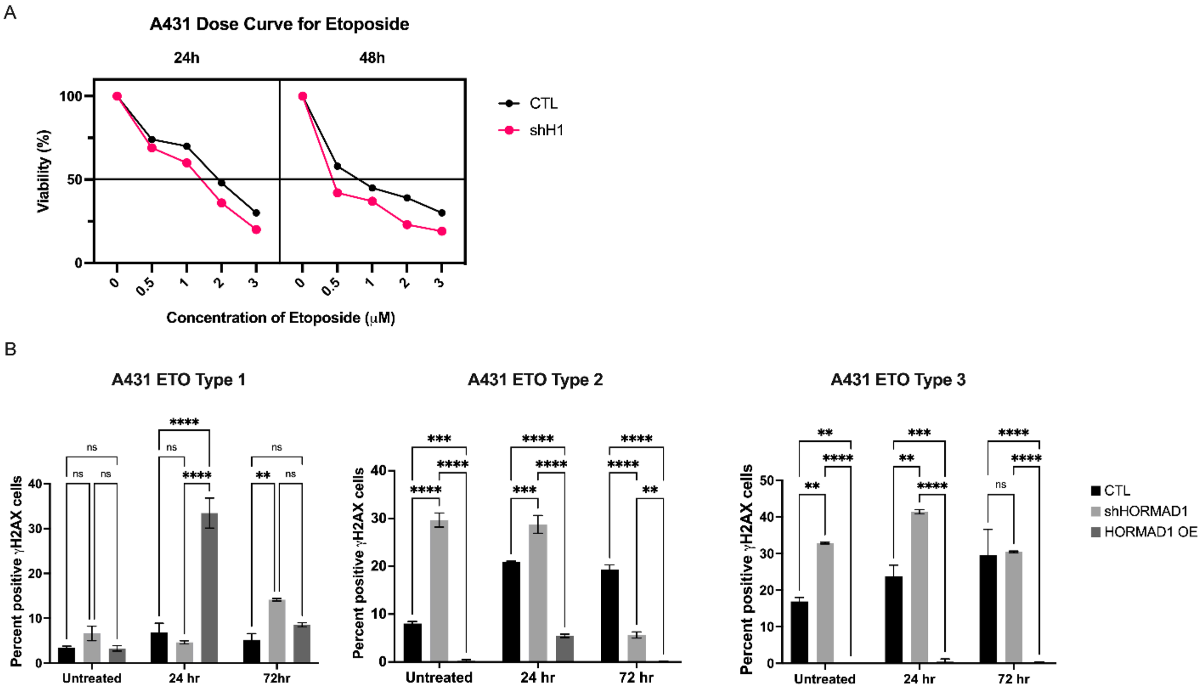

Figure S4. HORMAD1 knockdown increases sensitivity to etoposide. (A) A431 viability with increasing doses of etoposide as determined by an MTT assay (3-[4,5-dimethylthiazol-2-yl]-2,5-diphenyltetrazolium bromide). **(B)** Quantitative immunofluorescence cell count analysis demonstrating γH2AX staining by DNA damage type in control, shHORMAD1 and HORMAD1 OE A431 cells following 1μM etoposide treatment. Type 1 staining with <10 foci indicative of low DNA damage; type 2 with >10 foci indicative of high DNA damage; and type 3 with pan nuclear staining indicative of pre-apoptotic cells. HORMAD1 overexpression following etoposide treatment sustains low DNA damage (type 1) up to 72 hours and does not result in high DNA damage (types 2 and 3 staining). In contrast, shHORMAD1 cells treated with etoposide demonstrate significantly higher levels of DNA damage and preapoptotic cells at both 24 hours and 72 hours post drug exposure when compared to control cells. Values are means $\pm$ SEM, n=3, ****p>0.0001, ***p>0.001, **p>0.01. ETO, (etoposide).

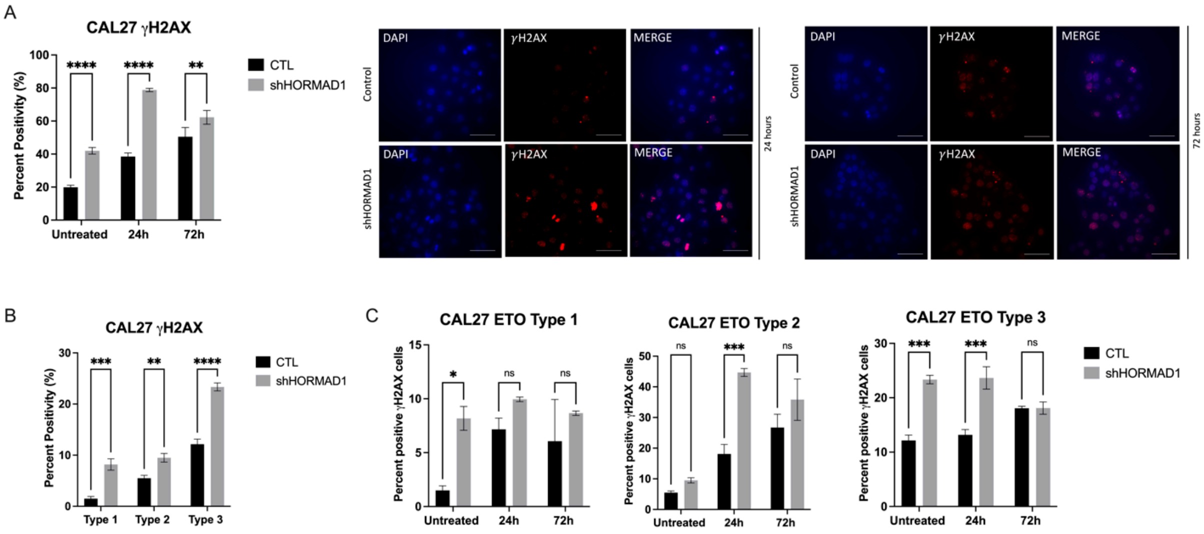

Figure S5. HORMAD1 depletion leads to high levels of DNA damage. (A) Quantitative immunofluorescence cell count analysis depicting γH2AX staining (red) in control and shHORMAD1 CAL27 cells treated with 1μM etoposide. Corresponding immunofluorescence at 24 hours and 72 hours following etoposide treatment. shRNA-mediated knockdown of HORMAD1 (shHORMAD1) in CAL27 cells results in a significant increase in γH2AX staining. Nucleic acid counterstained with DAPI (blue). Scale bars represent 50μm. **(B)** Distribution of percent γH2AX positive (red) cells according to DNA damage type (1, 2, 3) 24 hours following 1μM etoposide treatment. Type 1 with less than 10 foci indicative of low DNA damage; type 2 with more than 10 foci indicative of high DNA damage; and type 3 with pan nuclear staining indicative of pre-apoptotic cells. **(C)** Percent positive γH2AX cell distribution in untreated, 24-hours and 72-hours etoposide treated cells according to the type of staining. shHORMAD1 CAL27 cells experience significantly higher DNA damage (type 2) 24 hours following the treatment with etoposide. Additionally, shHORMAD1 cells exhibit an increase in preapoptotic staining in untreated cells and 24 hours following etoposide treatment indicating that shHORMAD1 knockdown renders CAL27 cells more sensitive to etoposide. Values are means ± SEM, n=3, ****p>0.0001, ***p>0.001, **p>0.01, *p>0.1. DDR (DNA damage response); ETO (etoposide).

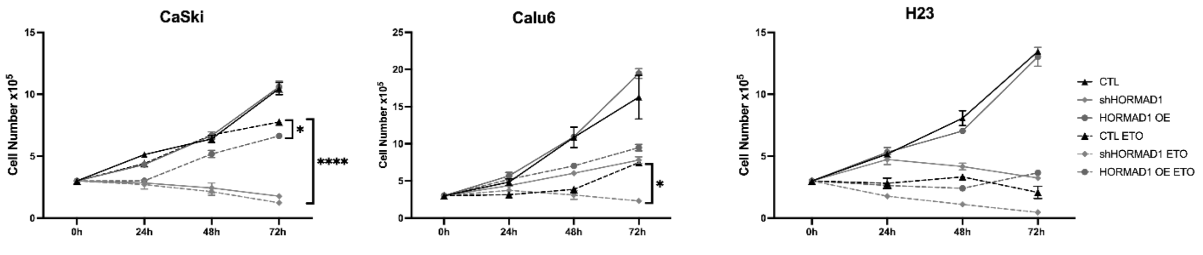

Figure S6. HORMAD1 expression decreases sensitivity to etoposide. Proliferation assays of cell lines representing cervical adenocarcinoma (CaSki) and lung adenocarcinomas (Calu6 and H23) reveal that shRNA mediated knockdown of HORMAD1 results in increased sensitivity to etoposide treatment compared to control and HORMAD OE cells. Values are means ± SEM, n=3, ****p>0.0001, *p>0.1.

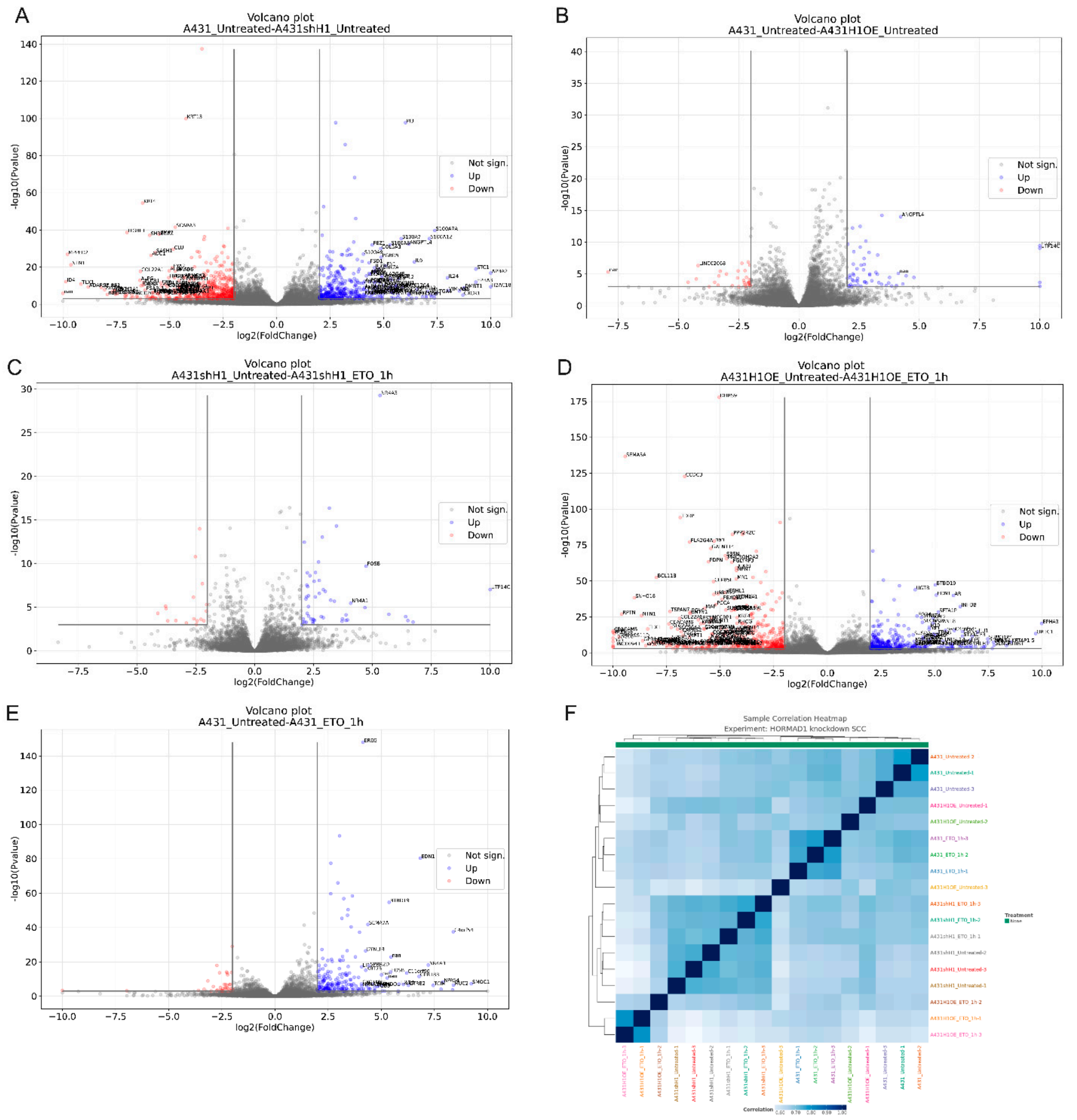

Figure S7. RNA-sequencing volcano plots of differentially expressed genes in treated and untreated A431 cells. The blue dots represent significantly upregulated genes, the blue dots represent significantly downregulated genes, and the grey dots represent insignificant differentially expressed genes. **(A)** A431 control vs. A431 HORMAD1 knockdown (shH1 represents shHORMAD1) cells; **(B)** A431 control vs. HORMAD1 overexpressing (H1OE) cells; **(C)** A431 shH1 (shHORMAD1) untreated vs. A431 shH1 etoposide treated cells; **(D)** A431 H1OE (HORMAD1 overexpression) untreated vs. A431 H1OE etoposide treated cells; **(E)** A431 untreated vs. A431etoposide treated cells; **(F)** Correlation heat map of RNA-seq A431 samples.

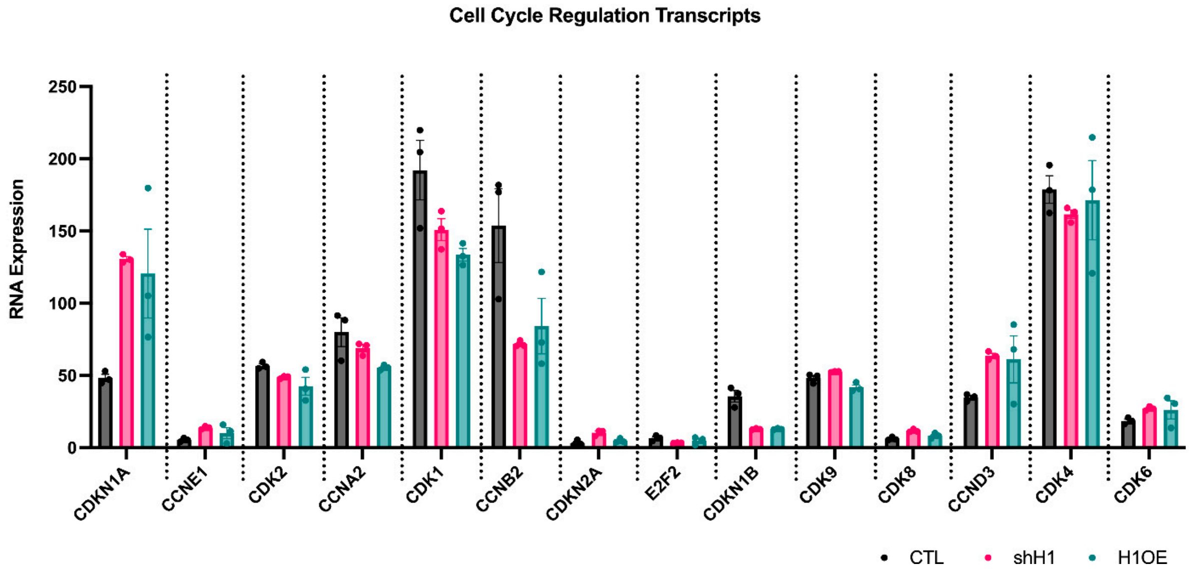

Figure S8. Cell cycle regulation RNA-sequencing transcript expression in HORMAD1 knockdown (shH1- shHORMAD1) and HORMAD1 overexpressing (H1OE) A431 cells compared to control (CTL) cells. RNA-sequencing transcript expression data of cell cycle regulation genes.

Gene construct	Dharmacon Catalogue No.	Sense Sequence	Hairpin Sequence
HORMAD1 #1	RHS4430-200207960	CTGCATCTTCTGAACTTGA	TGCTGTTGACAGTGAGCGACCTGCATCTTCTGAACTTGAATAGTGAAGCCACAGATGTATTCAAGTTCAGAAGATGCAGGGTGCCTACTGCCTCGGA
HORMAD1 #2	RHS4430-200189004	GTCCTTAATCTTGAGATAA	TGCTGTTGACAGTGAGCGCGGTCCTTAATCTTGAGATAAATAGTGAAGCCACAGATGTATTTATCTCAAGATTAAGGACCATGCCTACTGCCTCGGA
HORMAD1 #3	RHS4430-200289952	AGGAGCATTATACAAGTGA	TGCTGTTGACAGTGAGCGCCAGGAGCATTATACAAGTGATTAGTGAAGCCACAGATGTAATCACTTGTATAATGCTCCTGTTGCCTACTGCCTCGGA
HORMAD1 #4	RHS4430-200290988	AAGCAGTTTGTACACTAAA	TGCTGTTGACAGTGAGCGCAAAGCAGTTTGTACACTAAAATAGTGAAGCCACAGATGTATTTTAGTGTACAAACTGCTTTATGCCTACTGCCTCGGA
Non-Silencing Control	RHS4346		
GAPDH	RHS4430-200294325	AGTTGAGGTCAATGAAGGG	
STRA8 #1	RHS4430-200278659	TGTGCAAACACTCAAGTAA	TGCTGTTGACAGTGAGCGACTGTGCAAACACTCAAGTAAATAGTGAAGCCACAGATGTATTTACTTGAGTGTTTGCACAGCTGCCTACTGCCTCGGA
STRA8 #2	RHS4430-200284067	AGGAAGACAGTGTACTCTC	TGCTGTTGACAGTGAGCGCCAGGAAGACAGTGTACTCTCATAGTGAAGCCACAGATGTATGAGAGTACACTGTCTTCCTGATGCCTACTGCCTCGGA
STRA8 #3	RHS4430-200304957	GGAGAAGTTTCAGCTCTAT	TGCTGTTGACAGTGAGCGAAGGAGAAGTTTCAGCTCTATATAGTGAAGCCACAGATGTATATAGAGCTGAAACTTCTCCTCTGCCTACTGCCTCGGA
STRA8 #4	RHS4430-200305540	AGATCCTCTTTTTCAATCA	TGCTGTTGACAGTGAGCGAAAGATCCTCTTTTTCAATCAATAGTGAAGCCACAGATGTATTGATTGAAAAAGAGGATCTTGTGCCTACTGCCTCGGA
STRA8 #5	RHS4430-200304407	AGCTTAGAGGAGGTCAAGA	TGCTGTTGACAGTGAGCGACAGCTTAGAGGAGGTCAAGAATAGTGAAGCCACAGATGTATTCTTGACCTCCTCTAAGCTGCTGCCTACTGCCTCGGA
STRA8 #6	RHS4430-200197794	CTGTGCAAACACTCAAGTA	TGCTGTTGACAGTGAGCGCGCTGTGCAAACACTCAAGTAATAGTGAAGCCACAGATGTATTACTTGAGTGTTTGCACAGCTTGCCTACTGCCTCGGA

Table S1. HORMAD1 and STRA8 shRNA constructs. Construct 3 and 4 for HORMAD1 and constructs 1 and 3 for STRA8 were selected to perform experiments.
